# Profil des décès survenus chez les enfants âgés de 3 à 59 mois dans l’unité des soins intensifs d’un centre pédiatrique à Yaoundé-Cameroun

**DOI:** 10.11604/pamj.2020.36.246.11292

**Published:** 2020-08-05

**Authors:** Félicitée Nguefack, Evelyn Mah, Mina Ntoto Kinkela, Thierry Tagne, David Chelo, Roger Dongmo, Paul Koki Ndombo

**Affiliations:** 1 Faculté de Médecine et des Sciences Biomédicales, Université de Yaoundé I, Yaoundé, Cameroun,; 2 Hôpital Gynéco-obstétrique et Pédiatrique de Yaoundé, Yaoundé, Cameroun,; 3 Centre Mère et Enfant de la Fondation Chantal Biya de Yaoundé, Yaoundé, Cameroun,; 4 Institut Supérieur de Technologie Médicale, Yaoundé, Cameroun,; 5 Hôpital de District d’Efoulan, Yaoundé, Cameroun

**Keywords:** Décès, enfants, soins intensifs, causes, Death, children, intensive care, causes

## Abstract

**Introduction:**

le risque de décès serait élevé dans les unités des soins intensifs (USI) des pays en développement. Nous décrivons les décès survenus à l’Unité des Soins Intensifs du Centre Mère et Enfant de Yaoundé au Cameroun.

**Méthodes:**

étude rétrospective portant sur les caractéristiques cliniques, sociodémographiques, l’itinéraire thérapeutique ainsi que certains facteurs associés aux décès survenus entre 2010 et 2014 chez 200 patients âgés de 3-59 mois.

**Résultats:**

sur 2675 patients admis, 1807 étaient âgés de 3 à 59 mois et 303 sont décédés. Les taux de mortalité global et spécifique à cette tranche d’âge étaient de 11,3% et de 16,7% respectivement. La plupart (152/200 soit 76,0%) décédait à moins de 24 mois et le délai médian de leur admission était de 7 jours. Plus de la moitié (57,0%) avait recouru à un centre de santé et seuls 66 (33,0%) avaient bénéficié d’une référence. Le paludisme grave (41,5%), la pneumonie (22,7%) et la gastroentérite (27,8%) étaient les pathologies les plus incriminées. La malnutrition et le VIH/Sida constituaient les causes sous-jacentes de décès chez 23,0% et 20,5% de sujets respectivement. La présence de la gastroentérite multipliait le risque de décès d’environ 6 fois (OR = 5,76; P = 0,000) lorsque la malnutrition et l’infection à VIH étaient présentes. Les décès survenaient majoritairement (90,0%) dans les 72 heures d’admission.

**Conclusion:**

certaines pathologies auraient pu être traitées avec des moyens simples afin d’éviter les complications nécessitant une réanimation dans un contexte à ressources limitées. Il est crucial d’intensifier la lutte contre le paludisme, l’infection à VIH et la malnutrition.

## Abstract (en)

**Introduction:**

mortality risk is high at the Intensive Care Units (ICU) in developing countries. We here report the deaths occurred in the ICU at the Mother and Child Center in Yaounde, Cameroon.

**Methods:**

we conducted a retrospective study on the clinical, socio-demographic features, the therapeutic strategy as well as some of the factors associated with deaths occurred in 200 patients aged 3-59 months between 2010 and 2014.

**Results:**

out of 2675 patients included in the study, 1807 were aged 3-59 months and 303 died. The overall and cause-specific mortality rate in this age group was 11.3% and 16.7% respectively. Most patients (152/200; 76.0%) died within 24 months and the median admission time was 7 days. More than half of patients (57.0%) presented to a health center and only 66 (33.0%) presented to a referral hospital. Severe malaria (41.5%), pneumonia (22.7%) and gastroenteritis (27.8%) were the most common diseases. Malnutrition and HIV/AIDS were the underlying causes of death in 23.0% and 20.5% of patients respectively. Gastroenteritis multiplied the risk of death of approximately 6 times (OR = 5.76; p = 0.000) in patients affected by malnutrition and HIV infection. Deaths mainly occurred (90.0%) within 72 hours of admission.

**Conclusion:**

despite limited resources, some diseases could have been easily treated avoiding complications which require reanimation. It is essential to intensify the fight against malaria, HIV infection and malnutrition.

## Introduction

Bien que 40 millions de vies aient été épargnées dans le monde entre 1990 et 2015 [1], certains enfants continuent de mourir à une fréquence inacceptable. Les projections des décès vont dans le sens de l’aggravation si davantage d’interventions ne sont pas mises en œuvre pour les prévenir [2]. Le Cameroun n’a pas atteint la cible visée entre 1990-2015 qui était de 45 décès d’enfants de moins de 5 ans pour 1000 naissances vivantes, dans le cadre de l’Objectif du Millénaire pour le Développement n°4. Nombre de décès y sont enregistrés dans la tranche d’âge de 0 à 5 ans, soit 122 et 103 pour 1000 naissances vivantes [3]; une situation qui n’apparait pas isolée à ce seul pays [4]. Beaucoup de ces décès surviennent aussi bien lorsque les patients sont en communauté [5], que lorsqu’ils sont pris en charge dans les formations sanitaires. Le retard accusé par les familles dans la recherche des soins de santé adéquats se trouve à l’origine de certains [6]. Même lorsque les parents décident d’y recourir, habituellement le site choisi serait inapproprié pour les soins de qualité [7]. En effet et dans le cas de Yaoundé, il est établi que beaucoup de parents recouraient d’abord à l’automédication, à la médecine traditionnelle et aux centres de santé clandestins. Ce n’est qu’en dernier ressort qu’ils se rendraient vers une formation sanitaire de référence [6, 7]. Nombre de décès surviennent au cours du transport vers l’hôpital [7]. Les causes des décès survenant chez les enfants âgés de moins de 5 ans sont connues sur le plan mondial [2]. Ils succombent surtout de suite d’infections [8-10]; les mêmes circonstances se retrouveraient au Cameroun. La plupart peuvent être évitées par des mesures très simples [11, 12]. Beaucoup reste donc à faire pour relever les défis de l’accès aux soins afin d’améliorer la santé des enfants et d’atteindre les objectifs globaux de santé. Dans cette œuvre, les USI jouent un rôle majeur, lorsqu’adviennent des complications. Elles servent de relais d’autres services chargés de la mise en œuvre des soins primaires. Dans les USI, le taux de mortalité varie selon les différentes études [13, 14]. Les estimations du risque de décès à l’aide de l’index de mortalité s’élèveraient à 6% [14]. Notre étude décrit les caractéristiques des enfants admis et décédés dans l’USI d’un centre pédiatrique à Yaoundé. Elle s’attache, compte tenu des faits décrits, à rechercher les facteurs cliniques autres que le traitement, retrouvés chez les décédés.

## Méthodes

Il s’agissait d’une étude rétrospective conduite entre janvier et juin 2014 et qui a couvert la période allant du 1^er^ janvier 2010 au 31 décembre 2014. Elle s’est déroulée dans l’Unité des Soins Intensifs du Centre Mère et Enfant de la Fondation Chantal Biya (CME/FCB) de Yaoundé. Le CME/FCB, est une structure hospitalière qui a la plus grande fréquentation pédiatrique de la ville de Yaoundé. Il compte 9 unités et offre des services polyvalents avec le concours des pédiatres spécialisés dans divers domaines. Les pédiatres coiffent chacune des unités et sont assistés par des médecins généralistes et des infirmiers. Afin de réduire l’incidence des décès des enfants gravement malades, il y a été ouvert en 2005 une USI. Celle-ci a une capacité de 12 lits et la plupart des patients qui y sont admis proviennent soit d’autres formations sanitaires, des urgences pédiatriques du site, ou encore des autres unités d’hospitalisations. Nous y avons réalisé un échantillonnage consécutif des décès chez les sujets âgés de 3 à 59 mois. Ils devaient y avoir séjourné au moins une heure et bénéficié d’un début de prise en charge médicale. Les patients victimes de traumatisme accidentel et ceux qui avaient des pathologies tumorales étaient exclus de l’étude. Nous avons répertorié à partir des registres d’hospitalisation, les décès et colligé les informations des dossiers médicaux des décédés. Les données sociodémographiques, le mode de consultation, l’itinéraire thérapeutique, le délai d’admission, l’hospitalisation antérieure ainsi que la durée de séjour étaient analysés. Les données cliniques et le diagnostic présomptif ou de certitude évoqués par un pédiatre étaient en suite rapportés aux décès. Seuls 200 dossiers médicaux contenaient des informations exploitables et ont été retenus.

**Considérations éthiques:** notre étude a obtenu l’approbation du comité institutionnel d’éthique et de recherche de la Faculté de Médecine et des Sciences Pharmaceutiques de l’Université de Douala.

**Analyses statistiques:** les données étaient saisies dans Excel et analysées dans le logiciel épi info version 3.5.3. Nous avons exprimé les variables continues sous forme de médiane et intervalle interquartile. Les variables catégorielles étaient sous forme de proportions. Le test Khi^2^ a permis de comparer les proportions. À l’aide des analyses bivariées, nous avons décrit certaines variables explicatives susceptibles d’avoir influencé la survenue des décès. Lorsque la valeur de P était inférieure à 0,05, la différence entre les proportions était considérée significative.

## Résultats

### Fréquence des décès dans l’unité des soins intensifs

**Figure 1:**
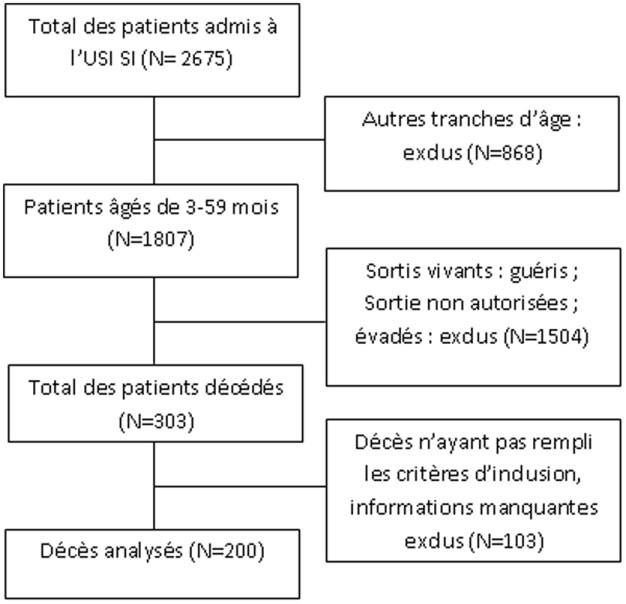
diagramme de recrutement des sujets décédés entre 2010 et 2014

Entre janvier 2010 et décembre 2014, 2675 patients étaient hospitalisés à l’USI. Les décès sont survenus chez 303 des 1807 âgés de 3-59 mois, soit 11,3% des patients admis et une mortalité spécifique de 16,7% pour la tranche considérée (Figure 1).

### Caractéristiques sociodémographiques

Près de 3/4 des sujets décédés (76,0%) avaient moins de 24 mois, leur âge médian était de 13 [IQE = 7-24] mois. Les garçons étaient plus nombreux (64,5%). L’âge médian de leurs mères était de 25 [IQE = 22-32] ans, la majorité avait entre 20-34 ans (67,0%) et était sans emploi (71,0%). Les rangs qu’occupaient les décédés correspondaient le plus souvent au chiffre de la fratrie (P = 0,000); la plupart (41,3%) était soit unique, ou occupait le 2^e^ rang. La moitié des décédés était issue d’une famille de 3 enfants [IQE = 2-5] (Tableau 1).

**Tableau 1:** caractéristiques générales de la population d’étude

Caractéristiques sociodémographiques		Effectif	Pourcentage (%)
Sexe	Masculin	129	64,5
	Féminin	71	35,5
Âge des enfants (mois)	3-11	80	40,0
	12-23	59	29,5
**Âge médian des enfants: 13 mois [IQE = 7-24 mois]**	≥ 24	61	30,5
Âge des mères (années) (N = 49)	< 20	9	19,0
	20-34	33	67,0
**Âge médian précisé pour les 49 mères: 25 ans [IQE = 22-32 ans]**	< 35	7	14,0
Profession des mères (N = 45)	Rémunérées	13	29,0
	Femmes au foyer	32	71,0
Fratrie (N = 196)	1-2	75	38,3
	3-4	57	29,0
**Nombre médian de la fratrie: 3 [IQE = 2-5]**	≥ 5	64	32,7
Rang des décédés (N =196)	1-2	81	41,3
	3-5	78	39,8
	≥ 6	37	18,9

### Recours aux soins

Les sujets étaient admis à l’USI en moyenne 10 jours après le début des premiers symptômes avec 39,0%, 7 jours après. La plupart (57,0%) avait recouru à un centre de santé et seuls 66 (33,0%) étaient référés. Par ailleurs, 21,0% des sujets qui avaient séjourné initialement dans une autre unité du CME/FCB provenaient du service de gastroentérologie et nutrition (Tableau 2).

**Tableau 2:** itinéraire thérapeutique des patients décédés

Variables		Effectif	Pourcentage (%)
**Référence d’autres formations sanitaires N = 66 (33,0%)**			
Type de formation sanitaire ayant référé le patient N = 66	Hôpitaux de district	19	29,0
	Centre de santé	38	57,0
	Hôpitaux de 1^er^ et 2^e^ catégorie	9	14,0
Patients hospitalisés à partir du service des urgences du site N = 158 (79,0%)			
Patients ayant séjourné dans un autre service du site avant le transfert interne vers l’USI N = 42 (21,0%)			
Unités du site où étaient hospitalisés les sujets avant l’admission à l’USI	Gastroentérologie et nutrition	18	43,0
	Infectiologie	13	31,0
	Cardiologie	8	19,0
	Néphrologie	3	7,0
Délai d’admission à l’USI (jours)	1-3	52	27,0
	4-7	64	34,0
	>7	74	39,0
Durée de séjour avant le décès à l’USI (heures)	n (%)		
	< 24	125	62,5
	24-72	55	27,5
	>72	20	10,0
USI = Unité de soins Intensifs			

### Pathologies

Les principaux diagnostics rapportés étaient le paludisme grave (41,5%), la pneumonie (22,7%) et la gastroentérite (27,8%). Les intoxications étaient moins en cause (4,3%) (Tableau 3). Certains sujets avaient en plus la malnutrition (23,0%) et le VIH/Sida (20,5%); plus de deux tiers (63,0%) de ceux qui étaient infectés par le VIH avaient la malnutrition et 43,6% la gastroentérite (Tableau 4). Les troubles métaboliques étaient notés seulement dans 29,0% des dossiers; ils étaient évoqués devant les convulsions fébriles et concernaient les hypoglycémies et hypocalcémies. Les sujets décédés en contextes de VIH et de malnutrition étaient les plus nombreux, soit 20 fois plus (OR = 20,18, P = 0,00) et le risque était multiplié par près de 6 lorsqu’il existait la gastroentérite (OR = 5,76; P = 0,00). Plus de 3/5 (63,0%) des malnutris étaient infectés par le VIH et 54,0% avaient la gastroentérite (Tableau 4). La malnutrition représentait une cause sous-jacente de décès dans 45,5% des cas de gastroentérite et dans 70,7% en cas d’infection à VIH/SIDA pédiatrique (Tableau 5).

**Tableau 3:** pathologies rapportées selon le mode d’admission dans le centre

Variables	Total N (%)	Sujets référés (N = 66 (33)	OR	IC ? 95%	P
Pathologies recensées					
Paludisme grave	83 (41,5)	33 (50,0)	1,69	0,93-3,08	0,057
Pneumonie	45 (22,7)	11 (16,7)	0,58	0,27-1,23	0,102
Gastroentérite fébrile	55 (27,8)	14 (21,2)	0,98	0,30-1,20	0,097
Malnutrition	46 (23,2)	7 (10,6)	0,28	0,12-0,67	0,001
Sida pédiatrique	41 (20,7)	7 (10,6)	0,34	0,14-0,82	0,009
Méningite	19 (9,6)	7 (10,6)	1,19	0,44-3,17	0,456
Anémie sévère	30 (15,2)	11 (16,7)	1,19	0,53-2,67	0,410
Sepsis sévère	19 (9,6)	6 (9,1)	0,91	0,33-2,51	0,537
Drépanocytose	7 (100)	2 (100)			
Insuffisance cardiaque	3 (1,5)	1 (1,5)	1,00	0,09-11,23	0,706
Causes directes de décès					
Choc septique	108 (71,5)	35 (61,4)	0,46	0,22-0,94	0,026
Déshydratation	26 (48,1)	17 (63,0)	3,40	1,11-10,40	0,028
Anémie sévère	31 (49,2)	12 (38,7)	0,43	0,16-1,19	0,082
Troubles métaboliques	18 (29,0)	4 (14,8)	0,26	0,07-0,92	0,028
Intoxications	2 (4,3)	2 (7,7)	-	-	-
Autres pathologies	8 (15,4)	4 (14,3)	0, 83	0, 19-3,76	0, 555

**Tableau 4:** effet de l’infection à VIH comme cause sous-jacente des décès selon la pathologie rapportée

Pathologies		Existence d’une infection à VIH		Effectif par pathologie	OR (IC à 95%)	P value
		Oui N (%)	Non N (%)			
Paludisme grave	Oui	12 (14,5)	29 (85,5)	83	0,51 (0,24-1,08)	0,038
	Non	71 (24,8)	88 (75,2)			
Gastroentérite	Oui	24 (43,6)	31 (56,4)	55	5,82 (2,80-12,15)	0,000
	Non	17 (11,7)	128 (88,3)			
Malnutrition	Oui	29 (63,0)	17 (37,0)	46	20,2 (8,72-46,75)	0,000
	Non	12 (7,8)	142 (92,2)			
Pneumonie	Oui	9 (19,6)	37 (80,4)	46	0,93 (0,40-2,12)	0,520
	Non	32 (20,8)	122 (79,2)			
Sepsis sévère	Oui	5 (26,3)	14 (73,7)	19	1,47 (0,50-4,38)	0,326
	Non	35 (19,4)	145 (80,6)			
Anémie sévère	Oui	6 (20,0)	24 (80,0)	30	0,96 (0, 36-2,54)	0,581
	Non	35 (20,6)	135 (79,4)			

**Tableau 5:** effet de la malnutrition comme cause sous-jacente des décès selon la pathologie rapportée

Pathologie		Existence d’une malnutrition		Effectif par pathologie	OR (IC à 95%)	P value
		Oui N (%)	Non N (%)			
Paludisme grave	Oui	13 (15,7)	70 (84,3)	83	0,47 (0,23-0,96)	0,027
	Non	33 (28,2)	84 (71,8)			
Gastroentérite	Oui	25 (45,5)	30 (54,5)	55	4,92 (2,43-9,94)	0,000
	Non	21 (14,5)	124 (85,5)			
Pneumonie	Oui	9 (19,5)	37 (80,5)	46	0,77 (0,34-1,74)	0,340
	Non	37 (24,0)	117 (76,0)			
Sepsis sévère	Oui	6 (31,6)	14 (68,4)	20	1,61 (0,57-4,52)	0,255
	Non	40 (22,2)	140 (77,8)			
Anémie sévère	Oui	8 (26,7)	22 (73,3)	30	1,26 (0,52-3,06)	0,378
	Non	38 (22,4)	132 (77,6)			
VIH/SIDA	Oui	29 (70,7)	12 (29,3)	41	20,19 (8,71-46,75)	0,000
	Non	17 (10,7)	142 (89,3)			

## Discussion

Nous avons relevé beaucoup de limites de la présente étude. Beaucoup de dossiers médicaux étaient exclus à cause du défaut d’informations. Les nouveau-nés et certains patients victimes de traumatismes physiques par accident ne sont pas pris en charge dans l’unité de soin intensif du site de cette étude. Par ailleurs, les patients souffrant des néoplasies nécessitent des soins prolongés avec des hospitalisations fréquentes. Leur prise en charge se fait dans des unités spécifiques. Leur admission dans l’USI exigerait un personnel formé pour des approches particulières notamment les soins palliatifs [15], qui ne seraient pas toujours observées en dehors de l’unité d’oncologie. Nous n’avons pas à cet effet inclus cette catégorie de patients dans nos analyses. Par conséquent, nos chiffres ne reflèteraient pas la réalité complète de la situation dans le site. Les décès n’ont pas été par ailleurs codifiés selon qu’il y ait eu échec à la réanimation cardio-pulmonaire, ou l’abstention à la réanimation, l’arrêt de l’acharnement thérapeutique ou encore la mort cérébrale. À cause des informations manquantes dans les dossiers médicaux, nous ne pouvions avoir les index de mortalité en soins intensifs [14]. En général ils ne sont pas estimés dans nos conditions de travail. Le taux de mortalité dans les services des soins intensifs varie selon les études; de 9,4% au Brésil [13] à 12,9% au Pakistan [16]. La mortalité dans notre site d’étude était élevée (16,6%) donc, un enfant âgé de 3 à 59 mois sur 6 décédait au décours des complications de sa maladie.

Des études ont montré que les décès en milieu hospitalier survenaient surtout dans les 48 heures suivant l’admission des patients [5, 17]. Dans un hôpital tertiaire, les enfants séjournaient pendant une durée médiane de 13 jours avant d’être envoyés au service des soins intensifs où ils mouraient [18]. Au Brésil près de 26,6% de patients mouraient 24 heures après l’admission aux soins intensifs [13]. Par ailleurs, les enfants qui séjournaient au-delà de 28 jours dans l’USI, avaient plus de risque de mourir [19]. Dans la présente étude, 9 décès sur 10 étaient enregistrés dans les 72 heures de l’admission, ceci reflèterait le profil général de décès au CME/FCB qui surviennent majoritairement (74,27%) dans les 48 heures [6]. Le retard dans la prise de décision de consulter dans une formation sanitaire expliquerait ces décès précoces. En effet, le recours aux soins était entrepris après au moins 4 jours de maladie chez plus de 1/3 (37%) des décédés. Cette décision de recours aux soins ne relèverait pas souvent des mères probablement du fait de leur dépendance financière vis-à-vis du conjoint ou d’une autre tierce personne. En effet, 71% étaient des femmes au foyer et par conséquent, leur capacité financière serait limitée. Il n’est donc pas rare que certains patients aient été soustraits de leur traitement même lorsque les chances de survie restaient élevées, situation souvent vécue dans nos hôpitaux. L’inaccessibilité financière et médicamenteuse expliquerait en partie cette situation. Dès lors, la relation entre la pauvreté et le recours aux soins est établie [20]. En effet, le coût direct de prise en charge de la pneumonie dans un contexte de VIH s’élèverait à US $ 435,12 en moyenne pour près de 8,67 jours [21]. À ceci s’ajoutent les coûts des services de soins intensifs qui sont exorbitants. Ces coûts seraient majorés lorsque surviennent diverses complications chez un même sujet.

Pour un pays comme le Cameroun où l’indice de pauvreté (37,5% en 2014) reste élevé [22], les coûts de prise en charge ne sont pas à la portée de plusieurs parents d’enfants admis en USI. Il n’existe pas encore un système de couverture universelle permettant à tous un accès équitable aux soins. Pour la plupart des populations de ce pays, l’accès aux soins est tributaire d’une imputation préjudiciable des moyens de subsistance de la vie courante. En Malaisie par exemple, ce type de paiement notamment de poche aurait occasionné des dépenses catastrophiques lors de la prise en charge des diarrhées [23]. D’ailleurs, le manque de moyens financiers avait déjà été évoqué par les parents de certains sujets décédés avant l’arrivée dans le site [7]. Un système de gratuité des soins primaires ou la mise en place d’une assurance maladie universelle permettrait d’éviter des états de santé graves occasionnés les obstacles financiers. Dans certains pays d’Afrique, l’exemption de paiement des soins a été mise en place afin d’améliorer l’offre de soins [24], cependant sa pérennisation serait problématique dans notre contexte.

Toutefois, d’autres obstacles notamment culturels avec la pratique de la médecine traditionnelle contribueraient également au retard aux soins [25]. Il en serait de même des soins inappropriés offerts aux malades dans les centres de santé clandestins qui pullulent dans le pays. Des auteurs ont démontré que plus de la moitié des décédés avaient recouru à une autre formation sanitaire avant l’admission dans leur site [26]. Dans la présente étude, 57,0% des décédés avaient été dans au moins un centre de santé clandestin communément désigné « GIC santé ». Il s’agit des groupements d’intervention communautaire érigés en centre de santé où sont délivrés fréquemment des soins irrationnels à but lucratif. Des errances diagnostiques ou thérapeutiques y seraient fréquentes [17]. La méconnaissance des signes de danger aussi bien par les familles et les personnels de ces structures aurait contribué à cette situation. À cet égard, le renforcement des compétences des familles et du personnel sur la Prise en Charge Intégrée des Maladies de l’Enfant (PCIME) est une nécessité.

Dans notre site, il n’existe pas un système d’estimations du risque de décès. Des auteurs ont évalué l’index de mortalité à 6% [14]. Sa cotation permet de prévoir les besoins en réanimation et de réfléchir sur les actions à entreprendre. Toutefois, la décision d’arrêt ou le refus d’amorcer une réanimation pédiatrique est une pratique courante [19, 27]. Des études ont relevé qu’à la demande des parents des gestes de réanimations n’auraient pas été entrepris chez certains patients [15, 28-30]. Il arrive aussi que les professionnels des USI renoncent au traitement palliatif surtout chez les patients qui ont séjourné longtemps [31]. Pourtant, les décisions de renonciation au traitement ou l’abstention à la réanimation doivent faire l’objet d’un consensus [32]. Elles seraient prises habituellement sans la concertation avec les familles [33].

Par ailleurs, les performances en réanimation du corps médical seraient relativement limitées. En effet, le plateau technique de notre site est inadapté à toutes les situations. Les kits de prise en charge des urgences n’existent pas. Les soins sont habituellement offerts après l’exécution des ordonnances remises aux parents. Le matériel, les médicaments et consommables nécessaires pour une réanimation irréprochable manquent souvent. Ceci est aggravé par les fréquentes rotations du personnel entre les unités et surtout de l’absence du recyclage des agents mutés. Par conséquent, la réanimation se limite souvent aux gestes d’aspiration des mucosités oropharyngées, à l’oxygénation des patients en détresse respiratoire et à la surveillance des paramètres vitaux, de l’état de conscience et de la diurèse. Le matériel indispensable à la respiration assistée n’existe pas alors que la plupart des sujets en aurait eu besoin.

La majorité des sujets admis à l’USI avec une malnutrition auraient besoin d’une ventilation assistée, souvent prolongée [34, 35]. Leur pronostic est grevé par l’existence de troubles métaboliques notamment l’hypoglycémie ou l’hyperglycémie à l’opposé des sujets bien nourris [36]. L’identification des troubles métaboliques n’est pas systématique chez tous les patients du fait des ruptures fréquentes en kits de diagnostic dans le site. Nous les avons notés dans seulement 29% de dossiers. Elles sont évoquées habituellement devant des convulsions sans fièvre. Bien que la malnutrition ne soit pas reconnue prédictive de la mortalité [34], nous l’avons trouvée fortement associée aux décès. D’ailleurs les statistiques des décès la sous-estiment comme cause de mortalité [37]. Pourtant elle contribuerait pour 56% de décès avec jusqu’à 83% attribués à la malnutrition modérée [37]. Par contre, la mortalité chez les enfants de moins de 5 ans est majoritairement attribuée aux causes infectieuses [8]. Dans notre étude, les pathologies infectieuses figuraient en bonne place et associées à la malnutrition sévère. Certains malnutris auraient acquis des infections nosocomiales car, 21% de sujets avaient séjourné dans d’autres unités du site avant d’être admis en réanimation. Les apports nutritionnels insuffisants favoriseraient l’acquisition de ces infections [35]. Ceci serait vrai dans notre contexte où il n’existe pas d’algorithmes de nutrition codifiés selon la gravité des désordres métaboliques.

En outre, le paludisme, la pneumonie et la gastroentérite étaient fréquemment retrouvées. L’infection à VIH aurait intervenu comme causes sous-jacentes. Toutes ces affections sont prises en compte dans le guide de PCIME. Sa mise en œuvre aurait permis de réduire la mortalité infanto-juvénile ailleurs [38]. Bien que les intoxications soient fréquemment retrouvées chez les patients hospitalisés aux soins intensifs pédiatriques, les décès à elles imputées sont peu fréquents [39, 40].

## Conclusion

Les décès précoces traduisaient la sévérité des pathologies dont la plupart était infectieuse. Certains sujets auraient pu être traités efficacement déjà à domicile et dans les formations sanitaires de premier recours avec des moyens simples et peu couteux si les directives de la PCIME étaient mises en œuvre. Il est nécessaire de renforcer le plateau technique dans l’USI en vue d’une réanimation effective des patients admis dans des états graves, facteur d’aggravation de l’incidence des décès précoces dans le site. Il est crucial d’intensifier la lutte contre le paludisme mais aussi, l’infection à VIH et la malnutrition.

### Etat des connaissances sur le sujet

- La prise en charge et la prévention des pathologies responsables des décès des enfants est possible avec les moyens simples et efficaces dans les pays en voie de développement;
- Le paludisme, les infections à VIH et la malnutrition continuent de faire les victimes parmi les jeunes enfants.

### Contribution de notre étude à la connaissance

- L’étude relève à travers l’itinéraire thérapeutique des patients, qu’il existerait un dysfonctionnement du système de référence et contre référence dans notre contexte;
- Elle présente la difficulté de prise en charge des enfants qui arrivent dans une unité de réanimation pédiatrique avec des pathologies évitables et qui en meurent soit parce que le délai d’admission était long, ou parce que le plateau technique n’aurait pas suffit pour une pris en charge adéquate.
